# Facile Fabrication of 3D Graphene–Silica Hydrogel Composite for Enhanced Removal of Mercury Ions

**DOI:** 10.3390/nano9030314

**Published:** 2019-02-27

**Authors:** Jinrong Lu, Xiaonan Wu, Yao Li, Yinghua Liang, Wenquan Cui

**Affiliations:** College of Chemical Engineering, Hebei Key Laboratory for Environment Photocatalytic and Electrocatalytic Materials, North China University of Science and Technology, Tangshan 063210, China; lujinrong@ncst.edu.cn (J.L.); uwxnyou@outlook.com (X.W.); liyao139020@gmail.com (Y.L.)

**Keywords:** silica gel, 3D graphene hydrogel, composite, adsorption Hg(II)

## Abstract

Adsorption is a highly promising and widely used approach to remove Hg(II) ions from contaminated water. The key to this technology is exploring the effective adsorbent. The three-dimensional (3D) graphene as reduced graphene oxide hydrogel (rGH)-encapsulated silica gel (SG-PEI/rGH) was prepared by a moderate chemical reduction strategy using ascorbic acid. This composite structure was characterized by FTIR, XRD, and SEM analysis and used as adsorbents for Hg(II) ions. Its adsorption capacity toward Hg(II) ions was 266 mg/g and increased about 32% compared with the silica gel because of reduced graphene oxide hydrogel (rGH). Mechanism study showed that the high adsorption ability was due to the formation of an N–Hg complex with multi-amino groups on the surface of polyethyleneimine-modified silica gel (SG-PEI) and the rapid diffusion of adsorbed ions attributed to the rGH network structure. This composite SG-PEI/rGH would be a promising material for the removal of Hg(II) ions.

## 1. Introduction

Along with fast industrial development, environmental pollution with heavy metals has increased drastically and become a global issue due to their toxicity [1]. Among the heavy metals, mercury is one of the most poisonous pollutants as it causes a variety of diseases by affecting neurological and renal activities. Released from industrial activities, such as coal combustion, gold mining, battery industry, chloralkali production, and so on, mercury pollutants are introduced into the water as Hg(II) ions [2,3]. Thus, it is necessary to exploit effective ways to remove Hg(II) ions from contaminated water.

Several methods, such as chemical precipitation, membrane filtration, ionic exchange, solvent extraction, and adsorption, have been employed for the separation of Hg(II) ions [4,5,6,7,8]. Among them, adsorption appears to be a highly promising and widely used approach due to high efficiency, low cost, operability, and repeatability [9,10,11]. The key to this technology is exploring the effective adsorbent. Several kinds of adsorbents have been employed, such as activated carbon [12], chelating resin [13], cellulose [14], metallic oxide [15], and silica gel [16]. Among these adsorbents, silica, gel-based material has attracted more interest in recent decades, because as an inorganic solid matrix, silica gel possesses characteristics such as well-modified surface, chemical stability, high specific areas, and environmental friendliness [17,18,19]. During the last few years, grafting chelation groups into the silica gel surface had been a major way to develop effective adsorbents based on silica gel. Ligand groups containing nitrogen, oxygen, and sulfur atoms have been employed to improve affinities for Hg(II) ions [20,21,22,23]. In particular, polyamine has been widely used as ligands due to its properties of high density of nitrogen as binding sites for Hg(II) [16]. However, there are still major challenges to develop silica gel-based adsorbents with high removal efficiency and transmission capability.

Recently, the development of multifunctional composite materials based on silica gel for the removal of Hg(II) has attracted considerable attention [24,25,26]. Graphene and graphene oxide have excellent performance in the adsorption of metal ions because of their large surface area, surface adsorption characteristics, and chemical stability [27,28,29]. They were combined with silica gel to form composite material as solid-phase extraction sorbents. Liu et al. linked graphene oxide sheets with silica gel via covalent bonds as high-performance adsorbents for solid-phase extraction [30]. Sereshti et al. reported a reduced graphene-modified 3-aminopropyltriethoxysilane (SiO_2_-APTES) composite used in the solid-phase extraction of Cr(VI) [31]. Feng et al. prepared reduced graphene oxide-encapsulated silica (SiO_2_@rGO) by the hydrothermal reduction strategy. This composite showed excellent adsorption capacity to chlorophenols (CPs) and peptides [32]. As we know, there was no composite composed of graphene and silica gel applied in adsorption of Hg(II). Herein, three-dimension (3D) graphene as reduced graphene oxide hydrogel (rGH) and polyamines-modified silica gel were integrated to combine their advantages. Firstly, the unique surface adsorption characteristics of graphene and its three-dimensional porous structure provide a fast channel for the movement of Hg(II) ions. On the other hand, polyamine-modified silica gel can be effectively coordinated with Hg(II) ions by multi-amino groups. With these considerations, a moderate reduction of GO and polyamine-polyethyleneimine modified silica gel (SG-PEI) mixtures were employed to fabricate 3D-graphene-encapsulated silica gel (SG-PEI/rGH), and the adsorption improvement of silica gel for Hg(II) was achieved. The composite was characterized by FTIR, SEM, and XRD analysis. The adsorption isotherms and kinetics properties were studied, and the adsorption mechanism was illustrated by XPS.

## 2. Materials and Methods

### 2.1. Synthesis of Polyethyleneimine(PEI)-Modified Silica Gel (SG-PEI)

The synthesis route of SG-PEI was shown in Scheme S1 (see in Supporting Information). First, 3.0 g of active silica gel was suspended in 100 mL of dry toluene, and 3 mL (3-Chloropropyl)trimethoxysilane (CPTS) was added. Then, the mixture was refluxed and stirred in an N_2_ atmosphere for 12 h. The isolated solid by filtration was treated in a Soxhlet extraction apparatus using toluene and ethanol, respectively, for one day. Then, the solid was dried in vacuum at 55 °C to obtain SG-Cl. 

Next, 1.0 g of SG-Cl was dissolved in 20 mL methanol, and 20 g of polyethyleneimine (PEI) was added. The mixture was stirred under reflux for 10 h. Then, the viscous liquid was washed with water and dried in vacuum at 55 °C for 48 h to obtain the solid SG-PEI.

### 2.2. Preparation of Amino-Silica Gel and 3D-Graphene Composites (SG-PEI/rGH)

Graphene oxide was prepared from graphite powder through the modified Hummers method [33]. SG-PEI/rGH was prepared by a simple chemical reduction of SG-PEI/GO by ascorbic acid. Firstly, 200 mg SG-PEI was added into 20 mL GO suspension with a concentration of 1 mg/mL under sonication for 1 h. Then, 200 mg ascorbic acid was added into the mixture and was heated to 95 °C for 1 h to obtain the composited hydrogel. Then, it was separated from solution and washed with ultrapure water. Before it was used as a sorbent, it was freeze dried under vacuum for 24 h.

### 2.3. Adsorption Behaviors Measurement

#### 2.3.1. Adsorption Experiment

First, 20 mL of mercury ions was placed in solution as perchlorate salt, and 20 mg of the adsorbent was added and stirred at 25 °C for 24 h. Then, the solution was filtered and the rest of the Hg(II) ions in the solution were tested by atomic absorption spectrophotometer. The adsorption capacity (*q*, in mg g^−1^) was calculated through the following equation:

(1) $$q=\frac{(C_{0}-C_{e})V}{m}$$

*C*_0_ and *C_e_* respectively represent the initial and the equilibrium concentrations of Hg(II) ions (mg·L^−1^), *V* represents the volume of the testing solution (L), and *m*(g) is the amount of the adsorbent.

The effect of solution pH on the adsorption was carried out in the pH range of 1.0–6.0 containing 20 mL of mercury ions (400 mg/L). 

The effect of the initial Hg(II) concentration on the adsorption was performed with varying concentrations in the range of 300–420 mg/L in pH 4. Using the isotherm data and Langmuir and Freundlich models [34], the process of adsorption onto the sorbent was described. The equation of Langmuir and Freundlich adsorption models can be respectively written as:

(2) $$\frac{c_{e}}{q_{e}}=\frac{1}{q_{m}}c_{e}+\frac{1}{q_{m}K_{L}}$$

(3) $$\ln q_{e}=\frac{1}{n}\ln c_{e}+\ln K_{F}$$

*q_e_* (mg·g^−1^) is adsorption capacity of the equilibrium state, *c_e_* (mg·L^−1^) is the equilibrium concentration of metal ions, *q_m_* is the saturated adsorption capacity (mg·g^−1^), and *K_L_* (L·mg^−1^), *K_F_* (L·g^−1^), *n* are the Langmuir and Freundlich adsorption constants, respectively.

In the fitting isotherms, R^2^ refers to the Adj.R-Square. It represents the correlation between data and fitting curve. 

#### 2.3.2. Adsorption Kinetics

To measure the adsorption kinetics, the adsorbents (20 mg) were added to 20 mL of solution with a mercury ion concentration of 400 mg/L and stirred at 25 °C. Stirring started at zero time of the adsorption process. Then, the samples were taken at the same time interval, and the adsorption capacity was examined. The kinetic adsorption plots were made according to pseudo-first-order and pseudo-second-order kinetic models. The pseudo-first-order kinetic model was:

(4) $$\ln(q_{e}-q_{t})=\ln q_{e}-k_{1}t$$

The pseudo-second-order kinetic model was:

(5) $$\frac{t}{q_{t}}=\frac{1}{k_{2}q_{e}{}^{2}}+\frac{t}{q_{e}}$$

*q_e_* represents the equilibrium adsorption capacity (mg g^−1^), *q_t_* represents the adsorption capacity at time of *t* (mg·g^−1^), and *k*_1_ (min^−1^) and *k*_2_ (g mg^−1^·min^−1^) respectively represent the rate constants of first-order and second-order adsorption.

In the fitting equations, R^2^ refers to the Adj.R-Square. It represents the correlation between data and fitting curve.

## 3. Results and Discussion

### 3.1. Construction and Characterization of SG-PEI/rGH Composite

**Figure 1:**
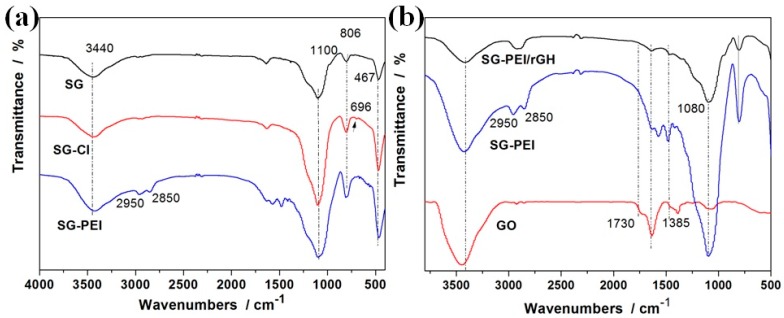
FTIR spectra of (**a**) SG-PEI and (**b**) SG-PEI/rGH composite.

SG-PEI was synthesized from activated silica gel (SG) via intermediate SG-Cl [35]. The modified SGs were characterized using FTIR spectroscopy. As shown in Figure 1a, the new bands appeared at 696 cm^−1^, corresponding to C-Cl vibration in the spectrum of SG-Cl which also retained the characteristic peaks at 3440 cm^−1^, 1100 cm^−1^, 974 cm^−1^, 806 cm^−1^, and 467 cm^−1^ of silica gel. After reacting with PEI, the band at 696 cm^−1^ of C-Cl band disappeared. Meanwhile the N-H bending vibration band at 1570 cm^−1^ and the asymmetric and symmetric vibration bands at 2850–2950 cm^−1^ of -CH_2_ appeared, indicating the successful modification of SG by PEI [36]. The results of elemental analysis showed the contents of C, H, and N in the SG-PEI, also confirming the successful grafting of PEI on the silica gel surface (Table S1). 

**Scheme 1:**
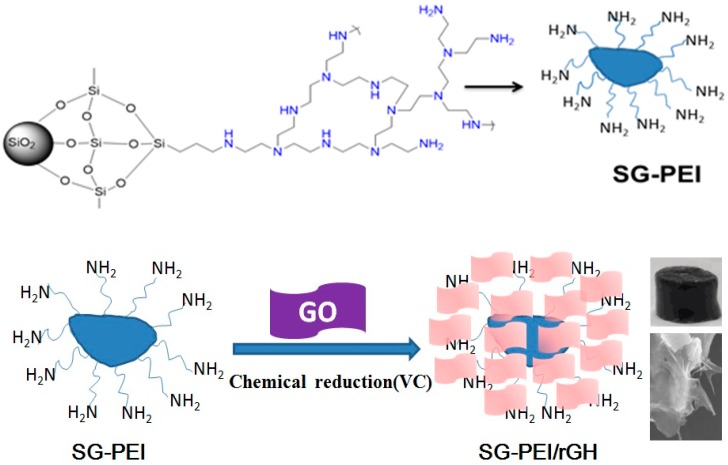
The synthesis route of polyamine-polyethyleneimine modified silica gel (SG-PEI)/reduced graphene oxide hydrogel (rGH) composite.

SG-PEI/rGH was prepared by a simple chemical reduction of SG-PEI/GO by ascorbic acid (Scheme 1). Firstly, SG-PEI and GO were mixed in aqueous solution under sonication, and they were combined well by the hydrogen bonding between NH_2_ groups of the SG surface and oxygen-obtaining groups such as carboxyl of GO. The hydrogen bonding interaction was proved by FTIR spectroscopy of SG-PEI/rGH in which the N-H and O-H bending vibration peak shifted to a lower wavenumber (Figure 1b). Then, GO was reduced by ascorbic acid to reduced graphene oxide hydrogel (rGH), and the SG-PEI was wrapped inside to form the NH_2_–SG/rGH composite which was confirmed by FTIR. Compared with GO, the peak at 1730 cm^−1^ of the oxygen functional group (C=O) in the spectrum of NH_2_–SG/rGH disappeared, which confirmed the successful reduction of GO [37]. Meanwhile, the characteristic peaks of SG-PEI suggest the existence of SG-PEI besides rGH in this composite. 

**Figure 2:**
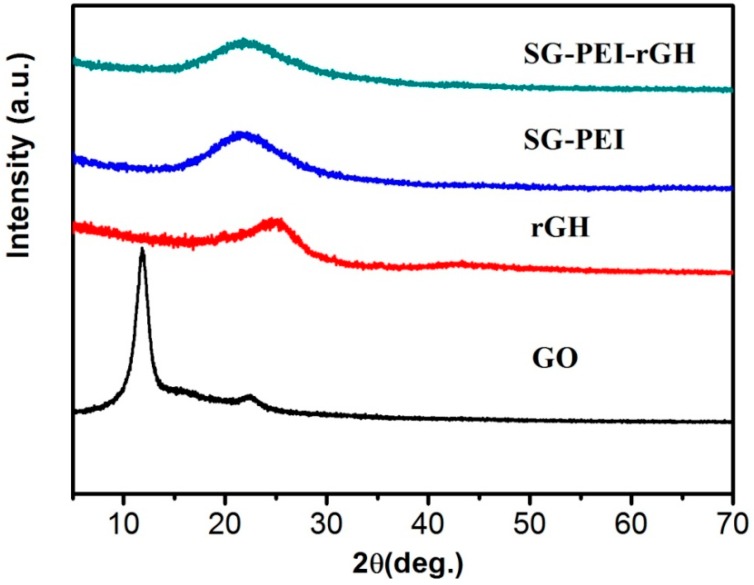
XRD patterns of GO, rGH, SG-PEI, and SG-PEI/rGH.

XRD was also determined to prove the reduction of GO in the composite. In the GO pattern, the diffraction peak at 12.1° corresponding to the interlayer spacing of 7.6 Å suggested the successful oxidation of graphite (Figure 2) [38]. When GO was reduced to rGH, the broad graphitic diffraction peak at approximately 25.1° appeared, and the diffraction peak at 12.1° disappeared. SG-PEI and SG-PEI/rGH showed amorphous structures, and a diffraction peak of rGH was not observed, which indicated that rGO sheets were disordered and rGH as a thin layer was dispersed on the NH_2_–SG surface [32]. In addition, there were no extra diffraction peaks in the spectrum, indicating that the third phase did not exist.

**Figure 3:**
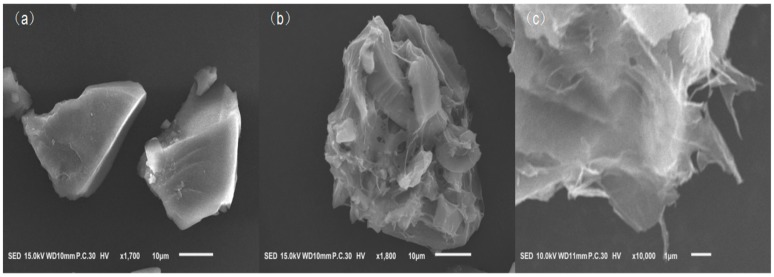
(**a**) SEM images of SG-PEI (**b**) SEM images of SG-PEI/rGH (**c**) the magnified SEM images of SG-PEI/rGH.

SEM was employed to characterize the morphology of the SG-PEI/rGH composite. It showed modified silica gel particles with an irregular shape (Figure 3a) and a rough surface that was covered with wrinkled gauze structures formed by rGH nanosheets (Figure 3b,c). rGH sheets extended beyond the edge of the SG-PEI, which significantly increased the adsorption site. In addition, rGH sheets also possessed a mesh structure and surface adsorption characteristic which reduce resistance for ion diffusion. The calculated BET (Brunauer−Emmett−Teller) specific surface area of SG-PEI was 182 m^2^/g, and when combined with rGH, the specific surface area was increased to 204 m^2^/g. Its higher specific surface area can promote the interaction with the adsorbed substance.

### 3.2. The Properties of Adsorption Hg(II)

#### 3.2.1. Static Adsorption Capacity and Adsorption Isotherms

**Figure 4:**
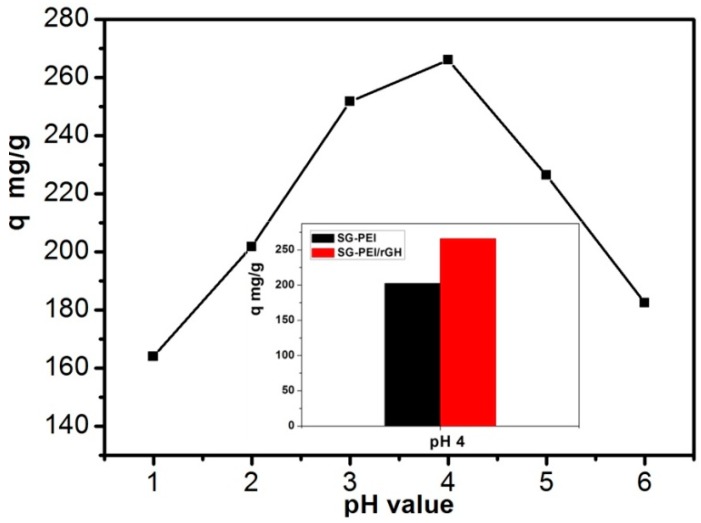
The effect of pH on adsorption capacity.

The adsorption of Hg(II) on the sorbents belongs to surface reaction because of functional groups such as the aminos on the surface of SG-PEI/rGH and because pH can affect its protonation. Thus, pH can affect the ability of adsorption of Hg(II) ions onto the adsorbents. The effect of solution pH on Hg(II) adsorption was studied in the range of 1.0–6.0 at 298 K. With the increase in pH value, the Hg(II) adsorption capacity of both adsorbents was increased until pH 4.0, and then it began to decrease (Figure 4). At pH 4, the adsorption capacity of SG-PEI/rGH was 266 mg/g, which exceeded most of the adsorbents reported [39], and for SG-PEI, it was 202 mg/g. In the low pH value, protons could compete with Hg(II) ions with the protonation of amino groups and occupy the active adsorbent sites. In addition, the Hg(II) adsorption capacity with SG-PEI/rGH was increased compared with that of SG-PEI. With the increased pH, the amino groups were free, and the competitive adsorption between H^+^ and Hg^2+^ was more weakened than that at low pH. As the pH continued to increase, the forms of Hg^2+^ changed to Hg(OH)^+^ or Hg(OH)_2_ when the mercury concentration was above 120 mg/L [40], causing the reduced adsorption capacity.

**Figure 5:**
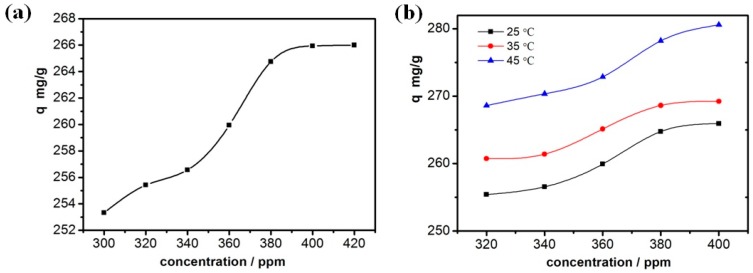
(**a**) The effect of Hg^2+^ initial concentration on adsorption quantity (**b**) The adsorption isotherm of Hg^2+^ at different temperatures.

**Figure 6:**
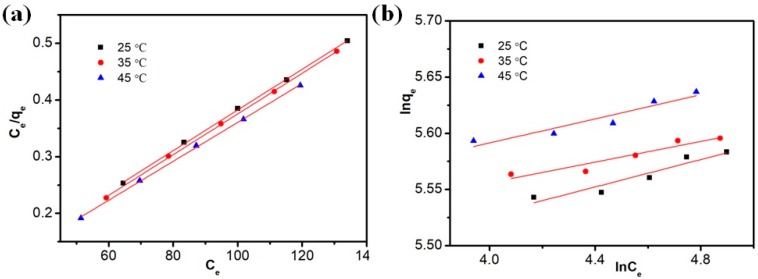
(**a**) Langmuir and (**b**) Freundlich isotherm for the sorption of Hg^2+^ onto SG-PEI/rGH at different temperatures.

The effect of initial Hg(II) ion concentration on the adsorption at 298 K was examined, and the results were shown in Figure 5a. It could be observed that the adsorption amount of Hg(II) increased as the initial Hg(II) concentrations gradually reached a platform, which was due to the saturation adsorption on the active sites of the SG-PEI/rGH. Figure 5b showed the adsorption isotherms of SG-PEI/rGH at different temperatures, such as 25 °C, 35 °C, and 45 °C. It was observed that as the temperature increased, the adsorption amount of Hg^2+^ also increased. The isotherm data were fitted according to the Langmuir and Freundlich equations. The fitted plots were shown in Figure 6 and the corresponding constants were in Table 1. It was revealed that the Langmuir equation well fitted the adsorption isotherm indicated by the more significant correlation than that of Freundlich equation (The coefficients and standard errors are listed in Table S2). In addition, the adsorption capacity of *q_m_* obtained by the Langmuir theory was 278 mg·g^−1^, which was close to the experimental value. This result suggested a monolayer adsorption of Hg(II) on SG-PEI/rGH.

To identify the thermodynamic properties of this adsorption process, thermodynamic constants such as Gibbs free energy (ΔG), enthalpy change (ΔH), and entropy change (ΔS) were determined (Figure S1) and shown in Table 2. ΔG was negative for the all-adsorption process at different temperatures, and the absolute values were increased as temperature was increased. It suggested that this adsorption was spontaneous and thermodynamically favorable [41]. The ΔH was positive, which indicated that this adsorption was endothermic, and the increased temperature was good for the adsorption process [23]. The thermodynamic results were consistent with experimental phenomena. 

#### 3.2.2. Adsorption Kinetics

**Figure 7:**
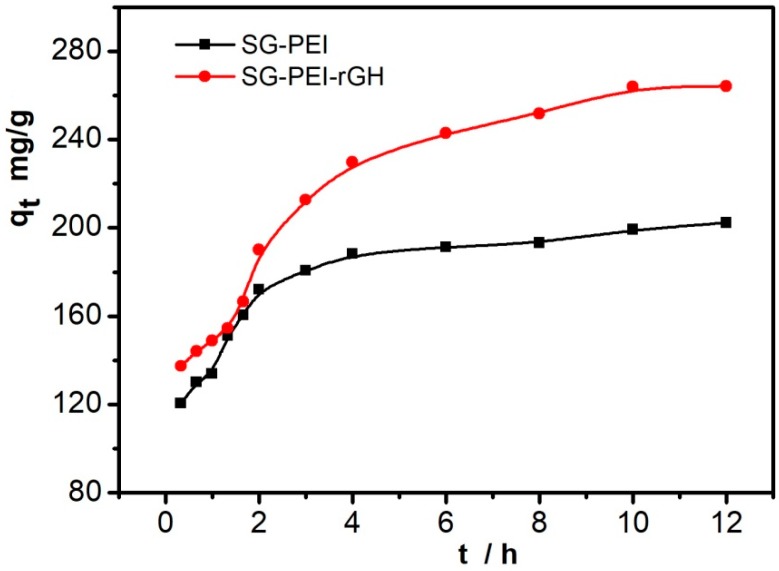
Kinetic sorption plot of Hg^2+^ on SG-PEI and SG-PEI/rGH.

The adsorption kinetics of Hg(II) by SG-PEI and SG-PEI/rGH were shown in Figure 7. For the two adsorbents, the adsorption rates of Hg(II) were both rapidly increased in the early stage of the process, and the main adsorption amount was accomplished in 2 h. Then, the adsorption rate changed slowly and reached to equilibrium after 10 h. In contrast, for SG-PEI, about 90% of total adsorption amount was accomplished within 2 h, and for SG-PEI/rGH, within 2 h, only about 70% of total adsorption amount was accomplished. Compared with SG-PEI, the increase in adsorption capacity for SG-PEI/rGH was mainly due to the increase in adsorption in the later adsorption stage after 2 h. This may be due to the rGH network structure loaded on the SG-PEI surface, which was favorable for the rapid diffusion of adsorbed ions. Therefore, at a later stage, the adsorption capacity would continue to increase. 

**Figure 8:**
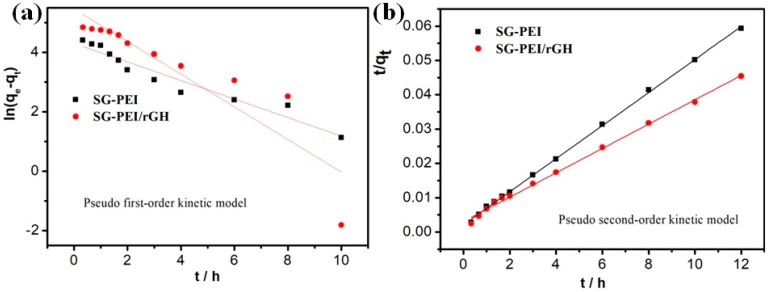
(**a**) Pseudo first-order kinetic model and (**b**) pseudo second-order kinetic model of SG-PEI and SG-PEI/rGH for Hg^2+^.

Pseudo-first-order and pseudo-second-order equations were employed respectively to fit the adsorption kinetic plots (Figure 8), and the corresponding kinetic constants for the two models are listed in Table 3 (Coefficients and standard errors of fitting equations are listed in Table S3). It showed that the correlation coefficients (R^2^) of the pseudo-second-order kinetic model for the two adsorbents were both higher than that of pseudo-first-order model, which means that these adsorption kinetics could be well described by the pseudo-second-order model and that this adsorption process was mainly controlled by chemisorption [42].

#### 3.2.3. The Effect of Coexisted Ions

**Figure 9:**
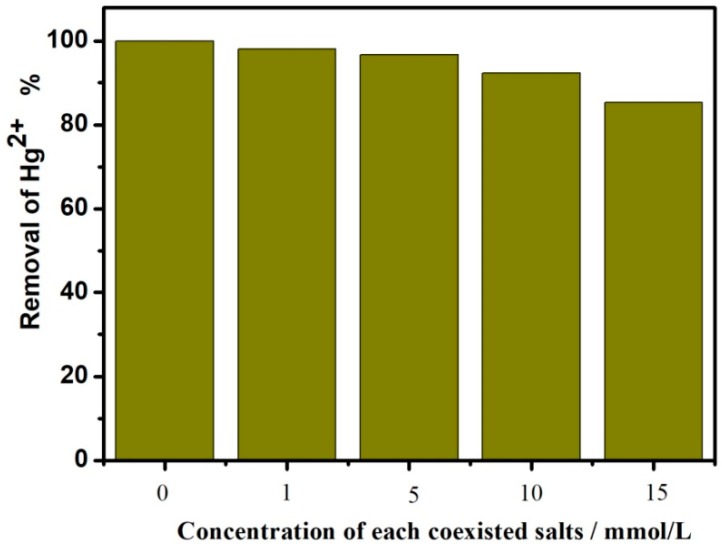
The effect of coexisting ion concentration on adsorption.

The coexisted cations in waste water would occupy adsorption sites of Hg(II) ions, hindering the adsorption process [43]. Therefore, common cations such as calcium, potassium, and sodium ions as nitrate salts were mixed with Hg(II) ions to examine their effects on adsorption of Hg(II) at the concentration range of 1–15 mmol L^−1^ for each cation. The adsorption results are listed in Figure 9, and it was observed that the salts had a slight inhibition to adsorption for Hg(II) ions, and when the concentration of Na^+^, K^+^, Ca^2+^, and NO_3_^−^ in solution was 15 mmol/L, 15 mmol/L, 15 mmol/L, and 60 mmol/L respectively, the removal rate can still reach over 85% without interference ions. Thus, the effect was relatively slight, indicating that SG-PEI/rGH as an adsorbent of Hg(II) showed excellent anti-interference to these ions in solution.

#### 3.2.4. Desorption and Adsorption Cycles

**Figure 10:**
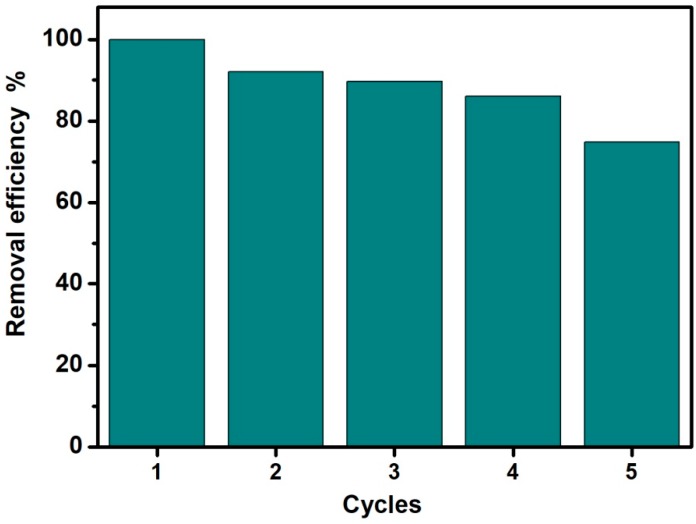
Adsorption–desorption cycle of SG-PEI/rGH for Hg^2+^.

The reusability of a sorbent is very important for its application, so the adsorption–desorption cycles were investigated (Figure 10). Dilute HCl solution (1 M) containing thiourea (2%) and cysteine (0.1 M) were chosen as the eluent to desorb loaded Hg(II) ions on SG-PEI/rGH. Then, to remove residual eluent, SG-PEI/rGH was washed with deionized water and employed for Hg(II) adsorption in the next cycles. It was observed that the adsorption efficiency was still about 75% after four cycles, which suggested that the SG-PEI/rGH showed good recycle capacity for Hg(II) adsorption.

#### 3.2.5. Mechanism of Adsorption Hg(II) Ions

**Figure 11:**
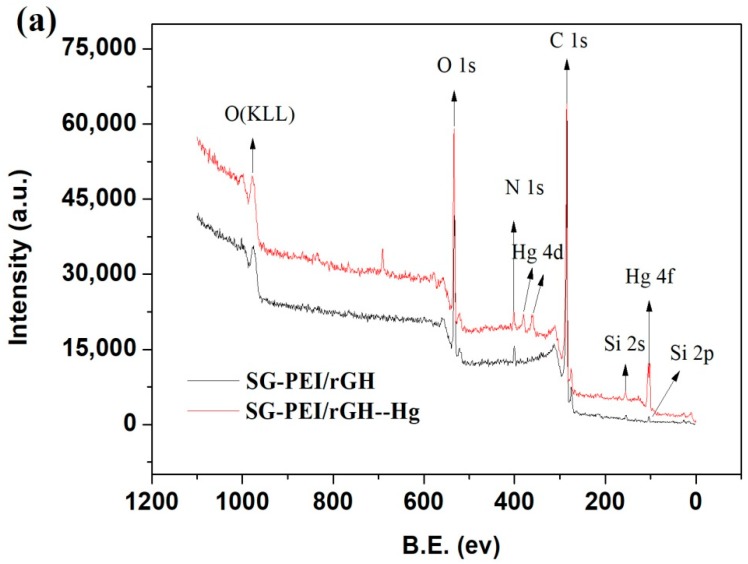

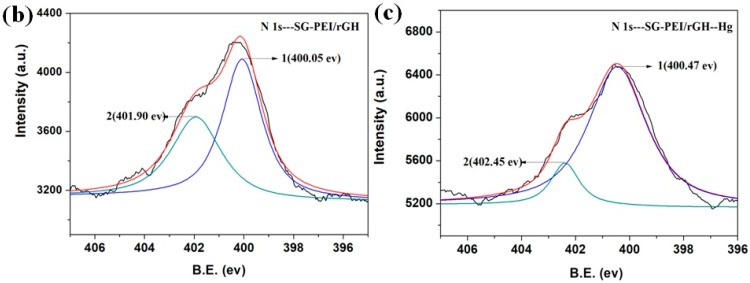
(**a**) XPS wide scan and (**b**,**c**) N1s spectrum of SG-PEI/rGH and SG-PEI/rGH-Hg.

**Figure 12:**
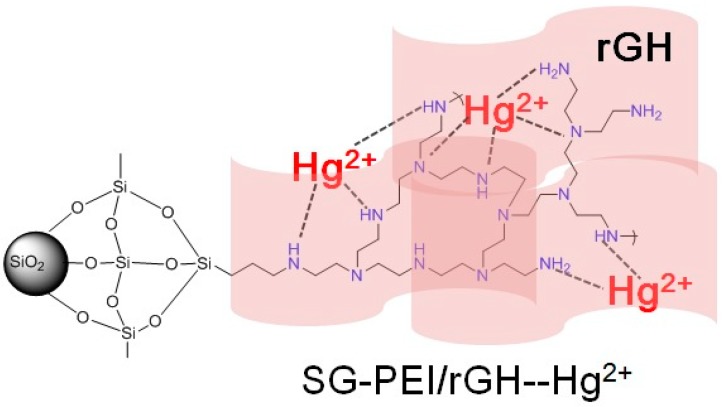
Mechanism for adsorption of Hg(II) on SG-PEI/rGH.

The X-ray photoelectron spectra (XPS) of SG-PEI/rGH were tested before and after adsorption Hg(II) ions to identify the interaction mechanism, and the results were shown in Figure 11. It was observed that after interaction with Hg(II) ions, the peaks of Hg 4d at 360 eV and 379 eV appeared, indicating that Hg(II) ions were loaded on the sorbent SG-PEI/rGH [44]. The spectra of N1s were resolved into two component peaks at 400.05 eV and 401.90 eV assigned to the N atoms in -NH/NH_2_ and -NR_2_ of PEI. After interaction with Hg(II) ions, they were shifted to 400.47 eV and 402.45 eV, respectively, which indicated the formation of N–Hg coordination [45]. Therefore, it was speculated that this adsorption process based on chemical adsorption was due to the strong coordination interaction of the N atom with Hg(II) (Figure 12). 

## 4. Conclusions

In summary, 3D, reduced graphene oxide hydrogel (rGH)-modified SG-PEI was prepared and employed in the adsorption Hg(II) ions. Compared with SG-PEI, it showed an improvement in adsorption capacity towards Hg(II) ions and operable recovery based on the presence of rGH. A kinetics and mechanism study showed that the high adsorption capacity was attributed to the formation of an N–Hg complex with multi-amino groups on the SG-PEI surface and the rapid diffusion of adsorbed ions because of the rGH network structure. Overall, SG-PEI/rGH would be a promising material for the removal of Hg(II) ions. Additionally, this work provides a new strategy for preparing 3D rGH-encapsulated silica, and it is expected that the composite material may also be extended to applications in other environmental purification processes.

## Figures and Tables

**Table 1:** Isotherm parameter of Langmuir and Freundlich for the adsorption of Hg^2+^ obtained by using linear method at different temperatures.

Torbent	T (K)	Langmuir			Freundlich		
		*K_L_* (L·mg^−1^)	*q_m_* (mg·g^−1^)	*R_L_* ^2^	*K_F_* (L·g^−1^)	*n*	*R_F_* ^2^
SG-PEI-rGH	298.15	0.1537	278.5515	0.99929	197.1569	16.4015	0.8872
	308.15	0.2209	278.5515	0.99963	215.2564	21.7250	0.8780
	318.15	0.1993	291.5452	0.99924	216.3332	18.6428	0.8753

**Table 2:** Thermodynamic parameters of Hg^2+^ adsorption on SG-PEI-rGH.

T	lnK	ΔG	ΔH	ΔS
(K)	-	(KJ·mol^−1^)	(KJ mol^−1^)	(J mol^−1^ K^−1^)
298.15	1.9695	−4.8820	9.1121	46.8698
308.15	2.0577	−5.2718		
318.15	2.2014	−5.8229		

**Table 3:** Kinetic parameter of the adsorption of Hg^2+^ onto SG-PEI and SG-PEI-rGH adsorbents.

Sorbent	T (K)	*q_e_*(exp) (mg·g^−1^)	Pseudo-First-Order Kinetics			Pseudo-Second-Order Kinetics		
			*k*_1_ (min^−1^)	*q_e_*(cal) (mg·g^−1^)	R_1_^2^	*k*_2_ (g·mg^−1^·min^−1^)	*q_e_*(cal) (mg·g^−1^)	R_2_^2^
SG-PEI	308.15	202.2859	0.3122	73.7359	0.9347	0.0109	207.4689	0.9993
SG-PEI-rGH	308.15	264.1632	0.5495	235.4244	0.8093	0.0041	281.6901	0.9966

## Supplementary Materials

The following are available online at https://www.mdpi.com/2079-4991/9/3/314/s1, Scheme S1: The synthesis route of SG-PEI from SG, Table S1: Elemental analysis of surface functionalized silica gel, Table S2: Coefficients and standard errors of fitting Langmuir and Freundlich isotherm, Table S3: Coefficients and standard errors of fitting Pseudo-first-order and pseudo-second-order equations, Figure S1: (a): lnK_d_ vs. C_e_ plot and (b) lnK vs. 1/T plot for the adsorption of Hg^2+^ on SG-PEI-rGH.
