# Supplementary material for: Facile Fabrication of 3D Graphene–Silica Hydrogel Composite for Enhanced Removal of Mercury Ions
Source: Nanomaterials (Basel). 2019 Feb 27;9(3):314. doi: 10.3390/nano9030314 (PMC6473818; doi:10.3390/nano9030314)
Supplement: Supplementary file 1 [file nanomaterials-09-00314-s001.pdf]

Facile Fabrication of 3D Graphene-Silica Hydrogel Composite for Enhanced Removal of Mercury Ions

Jinrong Lu, Xiaonan Wu, Yao Li, Yinghua Liang * and Wenquan Cui *

College of Chemical Engineering, Hebei Key Laboratory for Environment Photocatalytic and Electrocatalytic Materials, North China University of Science and Technology, Tangshan 063210, China

1. The Synthesis Route of SG-PEI

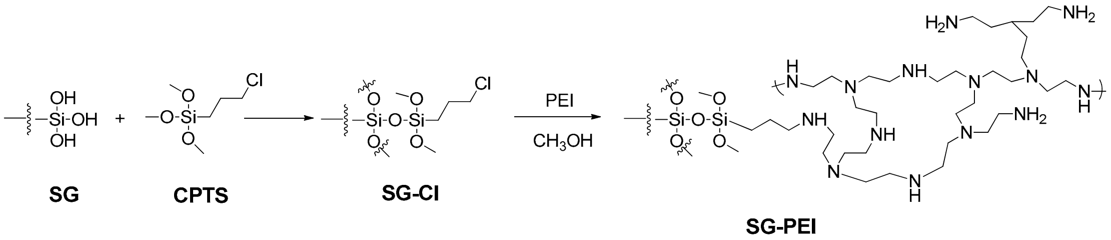

Scheme S1. The synthesis route of SG-PEI from SG.

2. Elemental Analysis

Table S1. Elemental analysis of surface functionalized silica gel.

Sample	Element Content (%)		
	C	H	N
SG-PEI	11.98	3.76	5.01

3. Coefficients and Standard Errors of Fitting Langmuir and Freundlich Isotherm

Table S2. Coefficients and standard errors of fitting Langmuir and Freundlich isotherm

T(K)	Langmuir					Freundlich				
	Intercept		Slopt		R^2	Intercept		Slopt		R^2
	Value	Error	Value	Error	R^2	Value	Error	Value	Error	R^2
298.15	0.02336	0.00489	0.00359	4.77196E−1	0.99929	5.282	0.04898	0.06097	0.0107	0.88715
308.15	0.01625	0.00341	0.00359	3.46935E−1	0.99963	5.37183	0.03817	0.04603	0.00844	0.87796
318.15	0.01721	0.00422	0.00343	4.734E−5	0.99924	5.37682	0.04398	0.05364	0.00995	0.87529

4. The Plot for Calculation of the Thermodynamic Constants

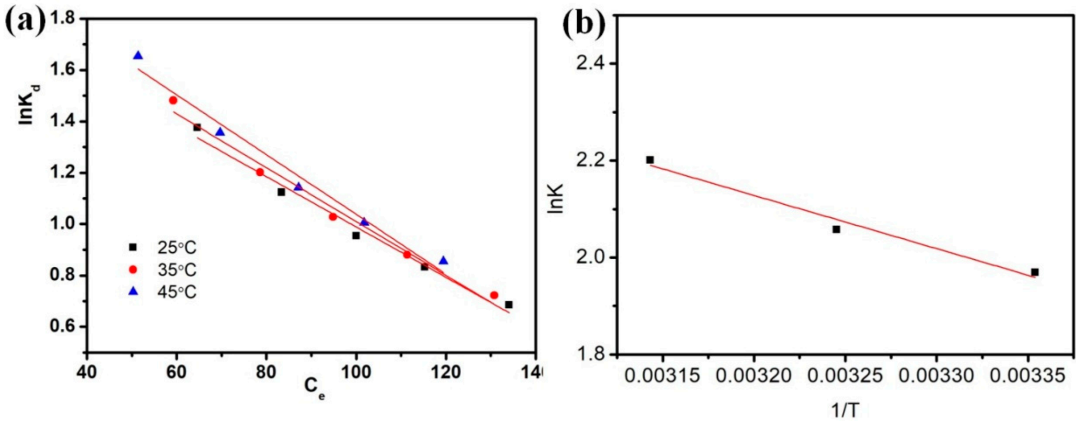

Figure S1. (**a**) lnK_d vs. C_e plot and (**b**) lnK vs. 1/T plotfor the adsorption of Hg^{2+} on SG-PEI-Rgh.

5. Coefficients and Standard Errors of Fitting Pseudo-first-order and Pseudo-second-order Equations

Table S3. Coefficients and standard errors of fitting Pseudo-first-order and pseudo-second-order equations

T (K)	Pseudo-first-order kinetics					Pseudo-second-order kinetics				
	Intercept		Slopt		R^2	Intercept		Slopt		R^2
	Value	Error	Value	Error	R^2	Value	Error	Value	Error	R^2
SG-PEI	4.30049	0.12023	−0.31224	0.026	0.93471	0.00213	2.14312E−1	0.00482	3.18289E−1	0.99931
SG-PEI-rGH	5.46139	0.38547	−0.54951	0.08338	0.80928	0.00308	3.52835E−5	0.00355	6.2774E−5	0.99656
